# GRMDA: Graph Regression for MiRNA-Disease Association Prediction

**DOI:** 10.3389/fphys.2018.00092

**Published:** 2018-02-20

**Authors:** Xing Chen, Jing-Ru Yang, Na-Na Guan, Jian-Qiang Li

**Affiliations:** ^1^School of Information and Control Engineering, China University of Mining and Technology, Xuzhou, China; ^2^School of Computer Science and Technology, Nankai University, Tianjin, China; ^3^College of Computer Science and Software Engineering, Shenzhen University, Shenzhen, China

**Keywords:** microRNA, disease, association prediction, graph regression, matrix factorization

## Abstract

Nowadays, as more and more associations between microRNAs (miRNAs) and diseases have been discovered, miRNA has gradually become a hot topic in the biological field. Because of the high consumption of time and money on carrying out biological experiments, computational method which can help scientists choose the most likely associations between miRNAs and diseases for further experimental studies is desperately needed. In this study, we proposed a method of Graph Regression for MiRNA-Disease Association prediction (GRMDA) which combines known miRNA-disease associations, miRNA functional similarity, disease semantic similarity, and Gaussian interaction profile kernel similarity. We used Gaussian interaction profile kernel similarity to supplement the shortage of miRNA functional similarity and disease semantic similarity. Furthermore, the graph regression was synchronously performed in three latent spaces, including association space, miRNA similarity space, and disease similarity space, by using two matrix factorization approaches called Singular Value Decomposition and Partial Least-Squares to extract important related attributes and filter the noise. In the leave-one-out cross validation and five-fold cross validation, GRMDA obtained the AUCs of 0.8272 and 0.8080 ± 0.0024, respectively. Thus, its performance is better than some previous models. In the case study of Lymphoma using the recorded miRNA-disease associations in HMDD V2.0 database, 88% of top 50 predicted miRNAs were verified by experimental literatures. In order to test the performance of GRMDA on new diseases with no known related miRNAs, we took Breast Neoplasms as an example by regarding all the known related miRNAs as unknown ones. We found that 100% of top 50 predicted miRNAs were verified. Moreover, 84% of top 50 predicted miRNAs in case study for Esophageal Neoplasms based on HMDD V1.0 were verified to have known associations. In conclusion, GRMDA is an effective and practical method for miRNA-disease association prediction.

## Introduction

MicroRNA (miRNA) is a small non-coding, single stranded and endogenous RNA molecule (containing 21~24 nucleotides) found in plants, animals, and some viruses, which functions in regulation of the gene expression by targeting mRNAs for cleavage or translational repression at the post-transcriptional level (Ambros, 2001, 2004; Bartel, 2004; Meister and Tuschl, 2004). The first miRNA was discovered in the early 1990s (Lee et al., 1993; Wightman et al., 1993). However, miRNAs were not recognized as a distinct class of biological regulators until the early 2000s (Pasquinelli et al., 2000; Reinhart et al., 2000; Lagos-Quintana et al., 2001; Lau et al., 2001; Lee and Ambros, 2001). Nowadays thousands of miRNAs from a wide variety of species have been found (Jopling et al., 2005; Kozomara and Griffiths-Jones, 2011). Furthermore, increasing researches have demonstrated that the miRNAs play crucial roles at multiple stages of the biological processes (Lee et al., 1993), such as early cell growth, proliferation (Cheng et al., 2005), differentiation (Miska, 2005), development (Karp and Ambros, 2005), aging (Bartel, 2009), apoptosis (Skalsky and Cullen, 2011), and so on. The dysregulation of the miRNAs has been confirmed as a main reason of aberrant cell behavior and important human complex diseases by many studies (Griffiths-Jones et al., 2006). More and more miRNAs have been verified to have associations with the development processes of many human diseases in experiments (Lynam-Lennon et al., 2009; Meola et al., 2009). For example, studies have implicated that epigenetic modulation of the miR-200 family has relevance to transition to a breast cancer stem cell-like state (Lim et al., 2013). Besides, recent study demonstrated that in human colorectal cancer cells, miR-186, miR-216b, miR-337-3p, and miR-760 could work in synergy to induce cellular senescence by targeting alpha subunit of protein kinase CKII (Kim et al., 2012). Therefore, identifying disease-related miRNAs is important and beneficial to treat, diagnose, and prevent human complex diseases. However, considering the huge amount of time and money we have to spend in carrying out experiments to verify a single miRNA-disease association, it is impossible to verify the associations one by one. Thus, it is necessary and valuable to choose the most likely associations to verify in the biological laboratory first. Therefore, considering there are some verified miRNA-disease datasets which can be treated as materials for prediction, we can develop computational models to rank and predict potential miRNA-disease associations.

In fact, scientists have already developed some computational methods in predicting miRNA-disease associations (Chen et al., 2012b, 2016c; Mork et al., 2014; Chen, 2016; Zeng et al., 2016; You et al., 2017). Many computational methods are based on a credible assumption that functionally similar miRNAs tend to have associations with phenotypically similar diseases to predict the potential associations between miRNAs and diseases. For example, Pasquier and Gardes (2016) constructed an vector space to predict miRNA-disease associations. They represented miRNA and disease distributional information with high-dimensional vectors respectively and then defined associations between miRNAs and diseases in terms of their vector similarity. Jiang et al. (2010) used a human phenome-microRNAome network to obtain the priority of miRNA-disease associations. Its weakness is that there was a high proportion of false positive and false negative samples in the miRNA-target interactions dataset on which this method extremely depended. To make up these weakness, a random walk algorithm-based model in protein-protein interaction (PPI) network was proposed (Shi et al., 2013). This method predicted potential associations between the miRNAs and diseases through combining the miRNA–target interactions, disease–gene associations, and PPIs. Mork et al. (2014) presented a miRNA-Protein-Disease Associations (miRPD) method which combined protein-disease association scores and miRNA-protein association scores to rank candidate miRNAs. Xu et al. (2014) introduced a systematic miRNA prioritization method based on known disease–gene associations and context-dependent miRNA-target interactions. Nonetheless, because of the high false positive and false negative samples existing in miRNA-target interactions and the incomplete disease-gene association network, the aforementioned methods could not provide sufficiently accurate prediction results.

Furthermore, based on the observation that miRNAs with similar functions are normally associated with similar diseases and vice versa, an effective prediction algorithm based on weighted *k* most similar neighbors for Human Disease MiRNAs prediction (HDMP) was proposed by Xuan et al. (2013) to predict the disease-related miRNAs using the miRNA functional similarity, disease semantic similarity, disease phenotype similarity, and the known miRNA-disease associations. However, the HDMP is not suitable to detect the association about a new disease which has no known related miRNAs. What's more, for a disease, if the number of its known related miRNAs is not enough, the prediction result will be not so satisfactory. Chen et al. (2012b) presented the first global network similarity-based computational model called Random Walk with Restart for MiRNA–Disease Association prediction (RWRMDA) by making use of the random walk algorithm based on the information of human miRNA functional similarity and known human miRNA–disease associations. RWRMDA obtained an excellent prediction performance. However, there is a non-negligible limitation that this method could not work for new diseases with no known related miRNAs. Chen et al. (2016b) proposed another model called Within and Between Score for MiRNA-Disease Association prediction (WBSMDA), which could effectively predict the potential miRNAs related to new diseases without any known related miRNAs and potential diseases related to new miRNAs without any known associated diseases. Recently, Chen et al. (2016c) developed a novel computational called Heterogeneous Graph Inference for MiRNA-Disease Association prediction (HGIMDA), using an iterative process with the initial probability vector, which can overcome the weakness that not being able to predict diseases with no known related miRNA, occurred in other methods.

Nowadays, machine learning has been applied in vast research fields and has great performance in many research problems (Chen et al., 2012a, 2016a,d; Huang et al., 2016; Zhang et al., 2017). Therefore, more and more studies have focused on using machine learning to solve problems of miRNA-disease association prediction. For instance, Xu et al. (2011) proposed a MiRNA-Target Dysregulated Network (MTDN) and constructed Support Vector Machine (SVM) classifier to identify positive miRNA-disease associations. However, since it is hard to obtain the negative miRNA-disease associations, the lack of negative samples would influence the accuracy of this method. Chen et al. (Chen and Yan, 2014) provided a method called Regularized Least Squares for MiRNA-Disease Association prediction (RLSMDA) which predicted potential disease-related miRNAs using semi-supervised learning method. RLSMDA could predict miRNAs associated with diseases without any known associated miRNAs and meanwhile it did not use negative associations between miRNAs and diseases. However, the choice of parameters for RLSMDA and the ways of combining the classifiers in different spaces together may influence prediction result to a large extent. Chen et al. (2015) further developed a computational model of Restricted Boltzmann Machine for Multiple types of MiRNA-Disease Association prediction (RBMMMDA), which can obtain both new miRNA-disease associations and their corresponding association types. Nevertheless, it is also difficult to make decision on the parameter values. Recently, Chen et al. (2017) proposed a method named Ranking-based KNN for MiRNA-Disease Association prediction (RKNNMDA) which integrated several trustable biological datasets to obtain a large data pool. However, this method may cause bias to those miRNAs that have more known associated diseases. Based on the fact that the miRNA-disease association matrix is low-rank, Li et al. (2017) presented Matrix Completion for MiRNA-Disease Association prediction (MCMDA). However, the optimal parameters of MCMDA are still in suspense.

In this study, we introduced a novel scoring method named Graph Regression for MiRNA-Disease Association prediction (GRMDA) to predict the potential miRNA-disease associations. We combined the Gaussian interaction profile kernel similarity and disease semantic similarity to get more complete integrated disease similarity. Integrated miRNA similarity was calculated in a similar way. We mapped three matrixes including miRNA-disease association matrix, integrated miRNA similarity matrix and integrated disease similarity matrix, into three graphs that were miRNA-disease association graph, miRNA similarity graph and disease similarity graph respectively. Then we synchronously applied graph regression on the three graphs, which involved three low-rank decompositions for projecting each of the three graphs into three latent spaces and two regressions between the three graphs. Finally, we got the scoring matrix by searching the minimum value of graph regression formula. Assuming that the five items of the formula are independent, we can calculate the minimum values of each item separately using Singular Value Decomposition (SVD) for low-rank decomposition and Partial Least-Squares (PLS) for graph regression. Furthermore, we used Leave-one-out cross validation (LOOCV) and five-fold cross validation to evaluate the effectiveness of GRMDA. As a result, GRMDA got an AUC of 0.8272 in LOOCV and obtained an average AUC with standard deviation of 0.8080 ± 0.0024 in five-fold cross validation. What is more, we applied three types of case studies to test the performance of GRMDA, including associated miRNA prediction for diseases based on known miRNA-disease associations from HMDD V2.0 database, for new diseases with no known related miRNAs and for the diseases based on known miRNA-disease associations from HMDD V1.0 database respectively. GRMDA performed well in the above validations and case studies, which means that GRMDA is practicable and effective in predicting potential miRNA-disease associations.

## Results

### Performance evaluation

**Figure 1:**
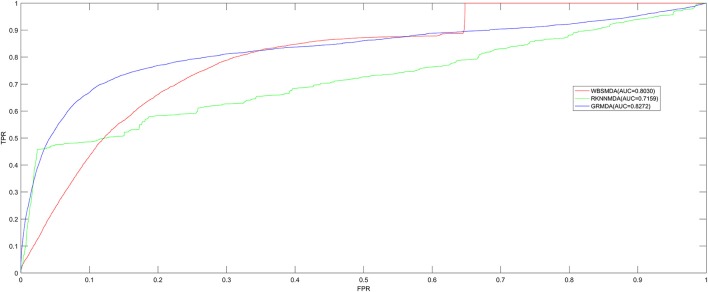
AUC of GRMDA in LOOCV compared with WBSMDA and RKNNMDA. As a result, GRMDA achieved AUC of 0.8272, which exceed the previous models.

We implemented LOOCV and five-fold cross validation to evaluate the performance of GRMDA. Both of LOOCV and five-fold cross validation are implemented using the recorded miRNA-disease associations in HMDD V2.0 database. During LOOCV, each one of the known miRNA-disease associations will be left out in turn to be considered as test sample. After calculating association scores of all the miRNA-disease pairs by GRMDA, we compared the score of the test sample with all the candidate pairs including all the miRNA-disease pairs which have no known associations to observe whether the rank of the test sample was above the threshold given in advance. Moreover, we plotted the true positive rate (TPR, sensitivity) vs. the false positive rate (FPR, 1-specificity) at different thresholds to obtain the Receiver operating characteristic (ROC) curves, which were shown in Figure 1. Sensitivity means the percentage of the positive samples correctly identified among all the positives; specificity refers to the percentage of negative samples correctly identified among all the negatives. Area under the ROC curve (AUC) is calculated as an index of the prediction ability of GRMDA, the value of which is between 0 and 1. Higher the AUC is, better the prediction performance will be. If AUC is smaller than 0.5, it means that the model performs not better than random prediction. As a result, GRMDA obtained the AUC of 0.8272 in the LOOCV as shown in Figure 1. The AUCs of WBSMDA and RKNNMDA in LOOCV are 0.8030, 0.7159 respectively. Therefore, according to the LOOCV results of these methods, we can intuitively observe the improvement of predicting the miRNA-disease associations with GRMDA.

During five-fold cross validation, we firstly randomly divided the known miRNA-disease associations into five parts with the same size. Then, each one of the five parts was treated as test samples in turn and the other four parts were treated as training samples. All of those miRNA-disease pairs that have no confirmed associations are candidate samples. After applying GRMDA, every score of test samples would be taken out to be compared with all the scores of candidate samples. Then we can get the rankings of test samples. In order to make the validation more accurate, we have repeated this procedure 100 times. Compared with RKNNMDA whose average AUC was 0.6723 ± 0.0027, average AUC of GRMDA in five-fold cross validation was 0.8080 ± 0.0024. The result confirmed that the GRMDA superior to RKNNMDA is able to predict miRNA-disease associations.

### Case studies

Based on two well-known miRNA-disease association databases, namely dbDEMC (Yang et al., 2010) and miR2Disease (Jiang et al., 2009), we studied Lymphoma to examine the practicability of GRMDA. In the end, we counted the number of the verified miRNAs in the top 10, top 20, and top 50 ones to evaluate the effectiveness of GRMDA.

Lymphoma is a group of blood cell tumors that develop from lymphocytes (a type of white blood cell) (Anagnostopoulos et al., 2000). According to the type of oncocyte, Lymphoma is divided into Hodgkin lymphoma (HL) and Non-Hodgkin Lymphoma (NHL) (Good and Gascoyne, 2008). About 90 percent of people who suffer Lymphoma have NHL (Alizadeh et al., 2000). Recent experimental studies showed the effect of re-expression of miRNA-150 on the formation of EBV-positive Burkitt lymphoma (Chen et al., 2013). A distinct set of five miRNAs (miR-150, miR-550, miR-124a, miR-518b, and miR-539) was shown to be differentially expressed in gastritis as opposed to MALT lymphoma (Thorns et al., 2012). After applying GRMDA method on Lymphoma, we got the result that 8 out of top 10, 17 out of top 20, and 44 out of top 50 potential miRNAs in the prediction result list for Lymphoma have been experimentally verified according to dbDEMC and miR2Disease (see Table 1). Compered with RKNNMDA and WBSMDA whose confirmed results are respectively 29 and 42 within top 50 predicted miRNAs for Lymphoma, GRMDA presents a more powerful predictive ability.

**Table 1:** Prediction of the top 50 predicted miRNAs associated with lymphoma based on known associations in HMDD V2.0 database.

**miRNA**	**Evidence**	**miRNA**	**Evidence**
hsa-mir-223	dbdemc	hsa-mir-34b	dbdemc
hsa-mir-125b	Unconfirmed	hsa-mir-29a	dbdemc
hsa-mir-34a	dbdemc	hsa-mir-128	dbdemc
hsa-let-7a	dbdemc	hsa-mir-23b	dbdemc
hsa-mir-9	dbdemc	hsa-mir-199b	dbdemc
hsa-mir-221	dbdemc; miR2Disease	hsa-mir-30a	dbdemc
hsa-mir-142	Unconfirmed	hsa-mir-222	dbdemc
hsa-mir-183	dbdemc	hsa-mir-106a	dbdemc; miR2Disease
hsa-mir-106b	dbdemc	hsa-mir-22	dbdemc
hsa-mir-195	dbdemc	hsa-mir-132	dbdemc
hsa-mir-182	dbdemc	hsa-mir-30e	dbdemc
hsa-mir-145	dbdemc; miR2Disease	hsa-mir-30d	dbdemc
hsa-mir-96	dbdemc	hsa-mir-141	dbdemc
hsa-let-7b	dbdemc	hsa-mir-335	dbdemc
hsa-mir-29b	dbdemc	hsa-mir-191	dbdemc
hsa-mir-181b	dbdemc	hsa-mir-194	dbdemc
hsa-mir-34c	Unconfirmed	hsa-mir-199a	dbdemc
hsa-mir-205	dbdemc	hsa-mir-15b	dbdemc
hsa-let-7c	dbdemc	hsa-mir-214	dbdemc
hsa-let-7e	dbdemc; miR2Disease	hsa-let-7f	dbdemc
hsa-mir-1	dbdemc	hsa-mir-27b	dbdemc
hsa-mir-146b	Unconfirmed	hsa-mir-103a	Unconfirmed
hsa-mir-143	dbdemc; miR2Disease	hsa-let-7i	dbdemc
hsa-let-7d	dbdemc	hsa-mir-429	Unconfirmed
hsa-mir-148a	dbdemc	hsa-mir-192	dbdemc

*The first column records top 1–25 related miRNAs. The second column records the top 26–50 related miRNAs*.

In order to help scientists to make use of our method and predictive results more efficiently, we have provided the prediction list of the whole potential miRNAs associated with all the human diseases and their association scores predicted by GRMDA (see Supplementary Table 1).

To estimate the applicability of GRMDA on the new diseases which do not have any known associations with miRNAs, we set all of the associations which involve the test disease as unknown ones. After implementing GRMDA, we obtained the ranking of the miRNA-disease association prediction scores. We use Breast Neoplasm as an example, the predicted result of which is shown in Table 2. From the result, we can see that 10, 20, and 50 related miRNAs out of the top 10, 20, and 50 have been confirmed by at least one of the three databases HMDD, dbDEMC and miR2Disease. The result that all the top 50 associations had been confirmed means that our method has a wonderful performance in this aspect. For example, hsa-mir-302b is ranked at top 1, which exhibits high frequency genomic alternations in human Breast Neoplasm (Zhang et al., 2006).

**Table 2:** Prediction of the top 50 predicted miRNAs associated with Breast Neoplasms based on known associations in HMDD V2.0 database by setting all of the associations which involve Breast Neoplasms as unknown ones.

**miRNA**	**Evidence**	**miRNA**	**Evidence**
hsa-mir-302b	dbdemc; HMDD	hsa-let-7c	dbdemc; HMDD
hsa-mir-302d	dbdemc; HMDD	hsa-mir-27b	dbdemc; HMDD
hsa-mir-181b	dbdemc; miR2Disease; HMDD	hsa-mir-96	dbdemc; miR2Disease; HMDD
hsa-mir-302a	dbdemc; HMDD	hsa-mir-195	dbdemc; miR2Disease; HMDD
hsa-mir-302c	dbdemc; HMDD	hsa-mir-298	HMDD
hsa-mir-338	dbdemc; HMDD	hsa-mir-339	dbdemc; HMDD
hsa-mir-135b	dbdemc; HMDD	hsa-mir-199b	dbdemc; HMDD
hsa-mir-149	dbdemc; miR2Disease; HMDD	hsa-mir-30b	dbdemc; HMDD
hsa-mir-106b	dbdemc; HMDD	hsa-mir-1	dbdemc; HMDD
hsa-mir-218	dbdemc; HMDD	hsa-mir-221	dbdemc; miR2Disease; HMDD
hsa-let-7f	dbdemc; miR2Disease; HMDD	hsa-mir-18a	dbdemc; miR2Disease; HMDD
hsa-mir-10b	dbdemc; miR2Disease; HMDD	hsa-mir-10a	dbdemc; HMDD
hsa-mir-210	dbdemc; miR2Disease; HMDD	hsa-mir-137	dbdemc; HMDD
hsa-mir-206	dbdemc; miR2Disease; HMDD	hsa-let-7b	dbdemc; HMDD
hsa-mir-708	HMDD	hsa-mir-20a	miR2Disease; HMDD
hsa-mir-187	dbdemc; HMDD	hsa-let-7d	dbdemc; miR2Disease; HMDD
hsa-let-7e	dbdemc; HMDD	hsa-mir-143	dbdemc; miR2Disease; HMDD
hsa-mir-516a	HMDD	hsa-let-7i	dbdemc; miR2Disease; HMDD
hsa-mir-219	dbdemc; HMDD	hsa-mir-101	dbdemc; miR2Disease; HMDD
hsa-mir-125a	dbdemc; miR2Disease; HMDD	hsa-mir-214	dbdemc; HMDD
hsa-mir-499a	HMDD	hsa-mir-663a	HMDD
hsa-mir-25	dbdemc; HMDD	hsa-mir-204	dbdemc; miR2Disease; HMDD
hsa-mir-19b	dbdemc; HMDD	hsa-mir-429	dbdemc; miR2Disease; HMDD
hsa-mir-152	dbdemc; miR2Disease; HMDD	hsa-mir-107	dbdemc; HMDD
hsa-mir-146b	dbdemc; miR2Disease; HMDD	hsa-mir-20b	HMDD

*The first column records top 1–25 related miRNAs. The second column records the top 26–50 related miRNAs*.

Finally, we used HMDD V1.0 to test GRMDA and observe whether our method has a good robustness by observing whether our method can keep a good performance in other dataset. According to the statistical results, we can see that 7, 16, and 42 respectively out of top 10, 20, and 50 miRNAs predicted to be related to the Esophageal Neoplasms have been confirmed by three databases mentioned above (see Table 3). For example, hsa-mir-196a which ranks the second in the top 50 has been confirmed that its binding-site SNP (rs6573) can regulate RAP1A expression, which contributes to the risk and metastasis of esophageal squamous cell carcinoma (Wang et al., 2012).

**Table 3:** Prediction of the top 50 predicted miRNAs associated with Esophageal Neoplasms based on known associations in HMDD V1.0 database.

**miRNA**	**Evidence**	**miRNA**	**Evidence**
hsa-mir-184	Unconfirmed	hsa-mir-125a	dbdemc
hsa-mir-196a	dbdemc; miR2Disease;HMDD	hsa-let-7d	dbdemc
hsa-mir-221	dbdemc	hsa-mir-150	dbdemc; HMDD
hsa-mir-19a	dbdemc; HMDD	hsa-let-7f	Unconfirmed
hsa-mir-99b	dbdemc; HMDD	hsa-mir-29b	dbdemc
hsa-mir-24	dbdemc	hsa-mir-34b	dbdemc; HMDD
hsa-mir-376a	dbdemc	hsa-mir-96	dbdemc
hsa-mir-301b	Unconfirmed	hsa-mir-188	dbdemc
hsa-mir-301a	dbdemc	hsa-mir-409	dbdemc
hsa-mir-449b	Unconfirmed	hsa-mir-140	dbdemc
hsa-mir-449a	Unconfirmed	hsa-let-7e	dbdemc
hsa-let-7a	dbdemc; HMDD	hsa-mir-302c	dbdemc
hsa-mir-203	dbdemc; miR2Disease;HMDD	hsa-mir-192	dbdemc;miR2Disease; HMDD
hsa-mir-299	dbdemc	hsa-let-7b	dbdemc;HMDD
hsa-mir-28	dbdemc; HMDD	hsa-mir-424	dbdemc
hsa-mir-222	dbdemc	hsa-mir-107	dbdemc; miR2Disease
hsa-mir-20b	dbdemc	hsa-mir-198	dbdemc
hsa-mir-144	dbdemc	hsa-mir-337	Unconfirmed
hsa-mir-495	dbdemc	hsa-mir-100	dbdemc; HMDD
hsa-mir-375	dbdemc; miR2Disease;HMDD	hsa-mir-130a	dbdemc; HMDD
hsa-mir-376c	Unconfirmed	hsa-mir-135a	dbdemc
hsa-mir-154	dbdemc	hsa-mir-491	dbdemc
hsa-let-7c	dbdemc; HMDD	hsa-mir-371	Unconfirmed
hsa-mir-20a	dbdemc; HMDD	hsa-mir-342	HMDD
hsa-mir-17	dbdemc	hsa-mir-186	dbdemc

*The first column records top 1–25 related miRNAs. The second column records the top 26–50 related miRNAs*.

In conclusion, the prediction performance of GRMDA is satisfactory. Because of that, we can foresee that with the development of experimental tools and the improvement of experimental measures, more and more miRNA-disease associations predicted by our method will be confirmed in the medical laboratory.

## Discussion

We introduced GRMDA in this paper, which was based on graph regression and similarity computational methods that integrates Gaussian interaction profile kernel similarity and disease semantic similarity or miRNA functional similarity. Because we introduced Gaussian interaction profile kernel similarity, the information of the disease similarity and the miRNA similarity was fully excavated to improve the accuracy of the prediction. To verify the accuracy of the GRMDA, we used LOOCV and five-fold cross validation and three case studies of human complex diseases. GRMDA has a good performance in all the above validations and case studies.

Here are the reasons why GRMDA has better performance than some previous methods. First of all, the miRNA similarity matrix and disease similarity matrix in GRMDA can take full advantage of the information from known miRNA-disease associations by introducing the Gaussian interaction profile kernel similarity, which means that miRNA-disease association matrix also takes part in the building of the above two similarity matrixes. In that way, GRMDA makes full use of the assumption that if two miRNAs affect the same disease, they tend to be similar (It is the same way for disease). Secondly, GRMDA applies Singular Value Decomposition (SVD) and Partial Least Squares Regression (PLS) during the graph regression to decompose a series of matrixes including association matrix, miRNA similarity matrix and disease similarity matrix. SVD and PLS are two modified forms of Principle Component Analysis (PCA) to collapse multidimensional data into low-dimension, which reconstruct the information of the original dataset with reduced components represented with vectors in latent spaces (Giuliani, 2017). In that way, our method can omit the less important attributes to avoid noise and pay attention to the more important attributes. For example, we will get three matrix *U*, Σ, and *V*^*T*^ after applying SVD on similarity matrix. Since Σ is a diagonal matrix in which every value represents the weight of an attribute about how significant the attribute affects the similarity between two miRNAs or two diseases, those small values can be abandoned to retain the most important attributes in the miRNA or disease similarity space. In the process of PLS, the principle components of latent association matrix and latent similarity matrix are extracted sequentially and then regressions are constructed between them with considering maximum correlation. In the end, because of our way of utilizing miRNA similarity matrix and disease similarity matrix, GRMDA could predict miRNAs for diseases with no one known related miRNA and predict diseases for miRNAs not related to any diseases, overcoming the limitations of some previous computational models.

GRMDA also has its weakness. Firstly, though current studies benefit from the increased known data, it is never a finished work to expand data which means our prediction is always under a data-lacking condition. Secondly, although GRMDA has an improvement in accuracy compared with other methods, the improvement is not enough. What's more, there are some difficulties in choosing parameters in SVD and PLS according to the size of the matrixes.

## Materials and methods

### Human MiRNA-disease associations

We downloaded data about human miRNA-disease associations between 383 diseases and 495 miRNAs from HMDD V2.0 database, which includes up to 5430 associations. The miRNA-disease association matrix *A* was built, where the entity *A*(*i,j*) will be 1 if miRNA *m*(*i*) and disease *d*(*j*) compose an association, otherwise 0. Variables *nm* and *nd* denote the number of miRNAs and diseases respectively.

### MiRNA functional similarity

The assumption that miRNAs with functional similarity tend to be associated with diseases that have phenotypical similarity is our basis to calculate the functional similarity score between miRNAs (Wang et al., 2010). Combining known miRNA-disease associations and disease similarity, the functional similarity between two miRNAs could be obtained through measuring the similarity between two disease sets that are related to the two miRNAs. The data came from http://www.cuilab.cn/files/images/cuilab/misim.zip, according to which we constructed the matrix *FS* to represent miRNA functional similarity, in which the entity *FS*(*m*(*i*)*, m*(*j*)) denotes the functional similarity score between miRNAs *m*(*i*) and *m*(*j*) with value from 0 to 1.

### Disease semantic similarity model 1

We use Directed Acyclic Graph (DAG) whose descriptor is *DAG*(*D*) = (*D,T*(*D*)*,E*(*D*)) to represent each disease, in which *T(D)* is the node set composed of node *D* itself and its ancestor nodes, *E(D)* is the edge set consisting of the direct edges from parent nodes to child nodes (Wang et al., 2010). The formula to calculate the semantic value of disease *D* is shown as below:

(1) $$\{\begin{array}{c} D1_{D}\left(d\right)=1\;if\;d=D\\ D1_{D}\left(d\right)=\max\left\{\Delta^{*}D1_{D}\left(d\prime \right)|d\prime \in \text{childrenof }d\right\}\;if\;d\neq D \end{array}$$

(2) $$DV1\left(D\right)=\sum_{d\in T\left(D\right)}D1_{D}\left(d\right)$$

Where Δ is the semantic contribution factor, whose value is between 0 and 1. For example, for a certain disease *D*, it contributes to the semantic value of itself with a value of 1. The farther the distance is from disease *D* to the disease *d* in *T*(*D*), the less the semantic contribution of *d* to *D* will be. Moreover, contributions from diseases in the same layer to the semantic value of disease *D* would be equal. The way to calculate semantic similarity between disease *d* (*i*) and *d* (*j*) comes from a reliable assumption that the larger part of the sharing of DAGs of two diseases, the larger the semantic similarity between them will be. The formula shown below is the semantic similarity between disease *d*(*i*) and *d*(*j*):

(3) $$\text{SS1}\left(\text{d}\left(\text{i}\right),\text{d}\left(\text{j}\right)\right)=\frac{\sum_{t\in T(d(i))\cap T(d(j))}\left(D1_{d\left(i\right)}\left(t\right)+D1_{d\left(j\right)}(t)\right)}{DV1\left(d\left(i\right)\right)+DV1(d(j))}$$

### Disease semantic similarity model 2

In this section, we calculated the disease semantic similarity following the method given in the reference (Xuan et al., 2013). The method in Disease semantic similarity model 1 has a good performance, however, it has a weakness. For example, if different diseases *d*_1_and *d*_2_are in the same layer of *DAG*(*D*), then as a result of disease semantic similarity model 1 defined in the section above, *d*_1_and *d*_2_ have the same contribution to the semantic value of disease *D*. But in certain circumstances, *d*_1_ may appear in less disease DAGs than *d*_2_. If that happens, it is easy to realize that *d*_1_ is more specific than *d*_2_ and should have a higher contribution to the semantic value of *D*. Therefore, we defined the contribution of disease *t* in *DAG*(*D*) to the semantic value of disease *D* as follows:

(4) $$D2_{D}\left(t\right)=-\log\left[\frac{the\;number\;of\;DAGs\;including\;t}{the\;number\;of\;diseases}\right]$$

The semantic value of disease *D* in model 2 is calculated in the similar way as in equation (2). The way to calculate semantic similarity between disease *d*(*i*) and *d*(*j*) also has same formation with method 1. The formulas are shown below:

(5) $$DV2\left(D\right)=\sum_{d\in T(D)}D2_{D}(t)$$

(6) $$SS2\left(d\left(i\right),d\left(j\right)\right)=\frac{\Sigma_{t\in T\left(d\left(i\right)\right)\cap T\left(d\left(j\right)\right)}(D2_{d\left(i\right)}\left(t\right)+D2_{d\left(j\right)}(t))}{DV2\left(d\left(i\right)\right)+DV2(d(j))}$$

*SS2* is the disease semantic similarity matrix calculated based on model 2 and its entity *SS2*(*d*(*i*)*,d*(*j*)) in row *i* column *j* is the disease semantic similarity between disease *d*(*i*) and *d*(*j*) based on disease semantic similarity model 2.

### Gaussian interaction profile kernel similarity for diseases

It is observed that functional similar miRNAs always tend to be associated with similar diseases. Based on this observation, we could utilize the topologic information extracted from the known miRNA-disease association network to compute the Gaussian interaction profile kernel similarity for diseases. First, we defined a binary vector *IP*(*d*(*i*)), the same value as the *i*th column of our miRNA-disease association matrix *A*, to represent the interaction profiles of disease *d*(*i*). The formula to calculate Gaussian interaction profile kernel similarity between disease *d*(*i*) and *d*(*j*) was shown below:

(7) $$KD\left(d\left(i\right),d\left(j\right)\right)=\exp\left(-\gamma_{d}{\left\|IP\left(d\left(i\right)\right)-IP(d(j))\right\|}^{2}\right)$$

(8) $$\gamma_{d}=\frac{{\gamma^{\prime }}_{d}}{\left(\frac{1}{nd}\sum_{i=1}^{nd}{\left\|IP(d(i))\right\|}^{2}\right)}$$

The effect of parameter γ_*d*_ is to control the kernel bandwidth. ${\gamma^{\prime }}_{d}$ is usually set as 1. γ_*d*_ is calculated by normalizing ${\gamma^{\prime }}_{d}$ by the average number of known miRNA-disease associations for all diseases. *KD* is the Gaussian interaction profile kernel similarity matrix for diseases.

### Gaussian interaction profile kernel similarity for miRNAs

MiRNA Gaussian interaction profile kernel similarity matrix is calculated in a similar way with disease Gaussian interaction profile kernel similarity:

(9) $$KM\left(m\left(i\right),m\left(j\right)\right)=\exp\left(-\gamma_{m}{\left\|IP\left(m\left(i\right)\right)-IP(m(j))\right\|}^{2}\right)$$

(10) $$\gamma_{m}=\frac{{\gamma^{\prime }}_{m}}{\left(\frac{1}{nm}\sum_{i=1}^{nm}{\left\|IP(m(i))\right\|}^{2}\right)}$$

*IP*(*m*(*i*)) has the same value with the *i*th row of our miRNA-disease association matrix *A* to denote the interaction profiles of miRNA *m*(*i*).

### Integrated similarity for miRNAs and diseases

In this work, the integrated disease similarity *S*_*d*_ were constructed based on disease semantic similarity *SS* and Gaussian interaction profile kernel similarity *KD*. Specifically, if disease *d*(*i*) and *d*(*j*) have semantic similarity, for simplicity, we assume that two types of semantic similarities between them are equally important. Then the final integrated similarity is computed directly using the average of *SS*1(*d*(*i*),*d*(*j*)) and *SS*2(*d*(*i*),*d*(*j*)), since both of two types of disease semantic similarity are calculated based on Directed Acyclic Graph (DAG) of diseases. Otherwise, if disease *d*(*i*) and *d*(*j*) do not have any semantic similarity, the integrated disease similarity equals to the Gaussian interaction profile kernel similarity which is taken as a supplement to the semantic similarity. The formula was shown as follows:

(11) $$\begin{array}{l} S_{d}\left(i,j\right)=\\ \;\{\begin{array}{l} \frac{\left(SS1\left(d\left(i\right),d\left(j\right)\right)\;+\;SS2\left(d\left(i\right),d\left(j\right)\right)\right)}{2}\;d\left(i\right)\text{ and }d\left(j\right)\text{ have}\\ \text{ semantic similarity}\\ \;KD\left(d\left(i\right),d\left(j\right)\right)\text{ otherwise} \end{array} \end{array}$$

Furthermore, by supplementing the miRNA functional similarity with miRNA Gaussian interaction profile kernel similarity, we obtained the integrated miRNA similarity as follows:

(12) $$\begin{array}{l} S_{m}\left(i,j\right)=\;\\ \;\{\begin{array}{l} FS\left(m\left(i\right),m\left(j\right)\right)\;m\left(i\right)\text{ and }m\left(j\right)\text{ have funtional similarity}\\ KM\left(m\left(i\right),m\left(j\right)\right)\text{ otherwise} \end{array} \end{array}$$

### GRMDA

**Figure 2:**
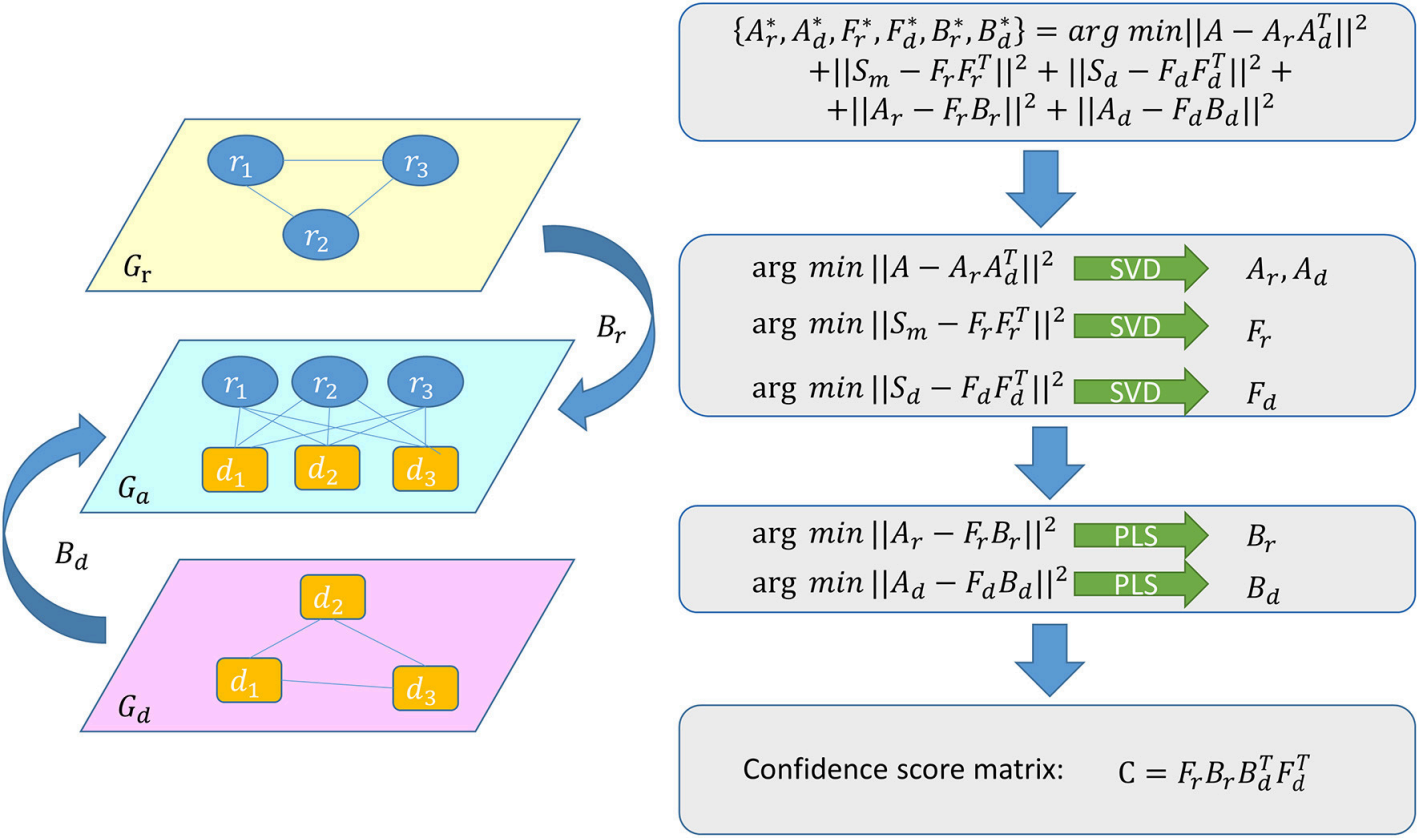
Flowchart of GRMDA model to predict the potential miRNA-disease associations based on the known associations in HMDD V2.0 database.

We use a graph regression (Hu et al., 2015) among *G*_*r*_, *G*_*d*_, and *G*_*a*_ which represent graphs about miRNA similarity network, disease similarity network and miRNA-disease association network respectively, to predict unknown associations between miRNAs and diseases (see Figure 2). Because the graph regression is synchronously performed in miRNA similarity space, disease similarity space and miRNA-disease association space, we can get the following formula:

(13) $$\begin{array}{l} \left\{A_{r}^{*},A_{d}^{*},F_{r}^{*},F_{d}^{*},B_{r}^{*},B_{d}^{*}\right\}\\ \;=\text{arg min}||A-A_{r}A_{d}^{T}|{|}^{2}+||S_{m}-F_{r}F_{r}^{T}|{|}^{2}\\ \;+||S_{d}-F_{d}F_{d}^{T}|{|}^{2}+||A_{r}-F_{r}B_{r}|{|}^{2}+||A_{d}-F_{d}B_{d}|{|}^{2} \end{array}$$

The first three items in formula (13) denote three low-rank decompositions to map *G*_*r*_, *G*_*d*_ and *G*_*a*_ in three spaces respectively. The first item helps to decompose *A* into two parts, each part represents the information of *G*_*a*_ in miRNA or disease aspect. The second item is used to convert *G*_*r*_ to feature matrix about miRNA and the third item is used in the same way for generating feature matrix about disease. The fourth item means a regression between miRNA-disease association space and miRNA similarity space, from which we can get the regression matrix which can connect *G*_*a*_ and *G*_*r*_. The fifth item represents a regression between miRNA-disease association space and disease similarity space, from which we can get the regression matrix which can connect *G*_*a*_ and *G*_*d*_. To be specific, we map the miRNAs and diseases in *G*_*a*_ into an *nm* × *r* miRNA associating matrix *A*_*r*_ and a *nd* × *r* disease associating matrix *A*_*d*_ respectively. We map miRNAs in *G*_*r*_ into an *nm* × *p* miRNA latent feature matrix *F*_*r*_. We map diseases in *G*_*d*_into a *nd* × *q* disease latent feature matrix *F*_*d*_. In the end, the *p* × *r* matrix *B*_*r*_ and the *q* × *r* matrix *B*_*d*_ are the corresponding regression coefficient matrices.

It is an intricate problem to minimize the objective function as a whole. However, since our goal is to make regression between the latent association spaces and similarity spaces, it is reasonable to divide the solution into two steps: obtaining the latent matrices and regressing between the latent matrices. To make the question easier, we assume that the five items in the formula (13) are independent. Then we can easily solve the above optimization problem by minimizing the items individually. We applied SVD for low-rank decompositions to generate *A*_*r*_, *A*_*d*_, *F*_*r*_, and *F*_*d*_ respectively in the following way:

(14) $$M\overset{SVD}{\Rightarrow }U\Sigma V^{T}=\left(U\sqrt{\Sigma }\right){\left(V\sqrt{\Sigma }\right)}^{T}=LR^{T}$$

SVD is an important and widely used method to decompose matrixes. SVD can condense the size of data and extract the possible association attributes between what the column and row represent. Σ is a diagonal matrix in which each value on the diagonal represents the importance of its mapped attribute. We can omit some attributes whose value is too small in Σ. However, if too small number of attributes are selected, the subtle information may be lost (Sharma, 2016). In this work, according to the particular data structure, we have retained about 45% (Franceschini et al., 2016) components for disease similarity space and miRNA similarity space. As a result, the parameters were set as *q* = 170 and *p* = 220 respectively. And for association space, the number of selected attributes was moderately set as *r* = 180. We operated a canonical correlation analysis on the principal components of miRNA latent feature matrix *F*_*r*_ and miRNA associating matrix *A*_*r*_, as well as disease latent feature matrix *F*_*d*_ and disease associating matrix *A*_*d*_ respectively to check for the mutual correlation between them. The results were shown in Supplementary Figures 1, 2. After that we utilized PLS regression on the latter two items in formula (13) to generate *B*_*r*_ and *B*_*d*_ individually. For the purpose to preserve the predictive ability with small noise in PLS (Kreeger, 2013), the percentage of components to keep was set as 90% both for regression between association space and miRNA similarity space and regression between association space and disease similarity space.

According to the previous formulas, we know that *F*_*r*_ represents the features of miRNA, *F*_*d*_ represents the features of diseases, *B*_*r*_ represents the relation between *A* and *F*_*r*_, *B*_*d*_ represents the relation between *A* and *F*_*d*_. Then, $B_{r}B_{d}^{T}$ builds a bridge between the features of miRNAs, the features of diseases and the associations between them. In the end, the confidence scores of miRNA-disease pairs to be potential associations are calculated in the following formula:

(15) $$C=F_{r}B_{r}B_{d}^{T}F_{d}^{T}$$

where, *C* is the confidence score matrix and *C*(*i,j*) represents the associations core of miRNA *m*(*i*) and disease *d*(*j*). The higher the score is, the more likely the association exists.

## Author contributions

XC conceived the project, developed the prediction method, designed the experiments, analyzed the result, and wrote the paper. J-RY implemented the experiments, analyzed the result, and wrote the paper. N-NG analyzed the result and revised the paper. J-QL analyzed the result. All authors read and approved the final manuscript.

### Conflict of interest statement

The authors declare that the research was conducted in the absence of any commercial or financial relationships that could be construed as a potential conflict of interest.
