# Supplementary figures and images for: GRMDA: Graph Regression for MiRNA-Disease Association Prediction
Source: Front Physiol. 2018 Feb 20;9:92. doi: 10.3389/fphys.2018.00092 (PMC5826195; doi:10.3389/fphys.2018.00092)

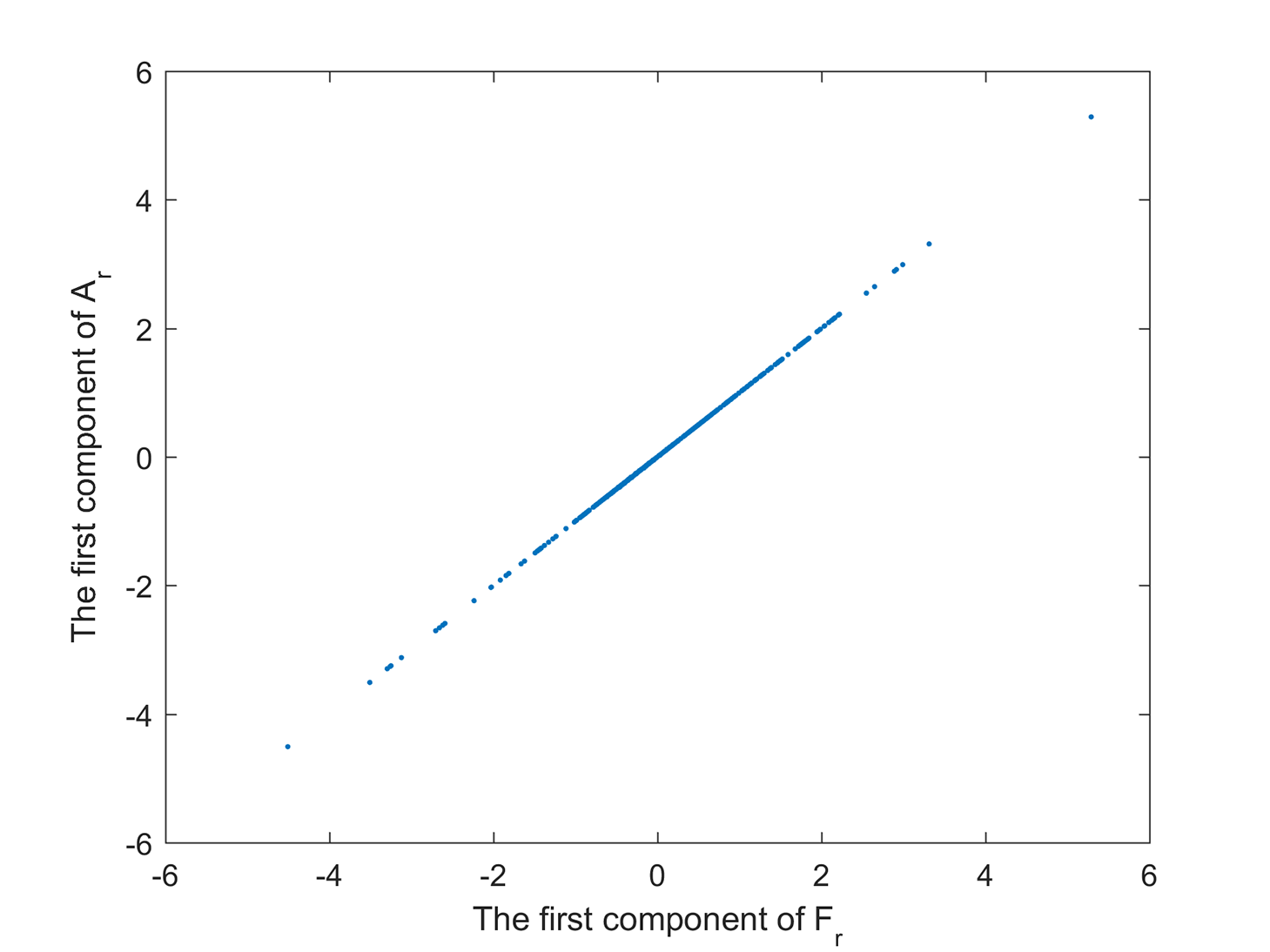

Supplement: Supplementary file 1 [file Image1.TIF]

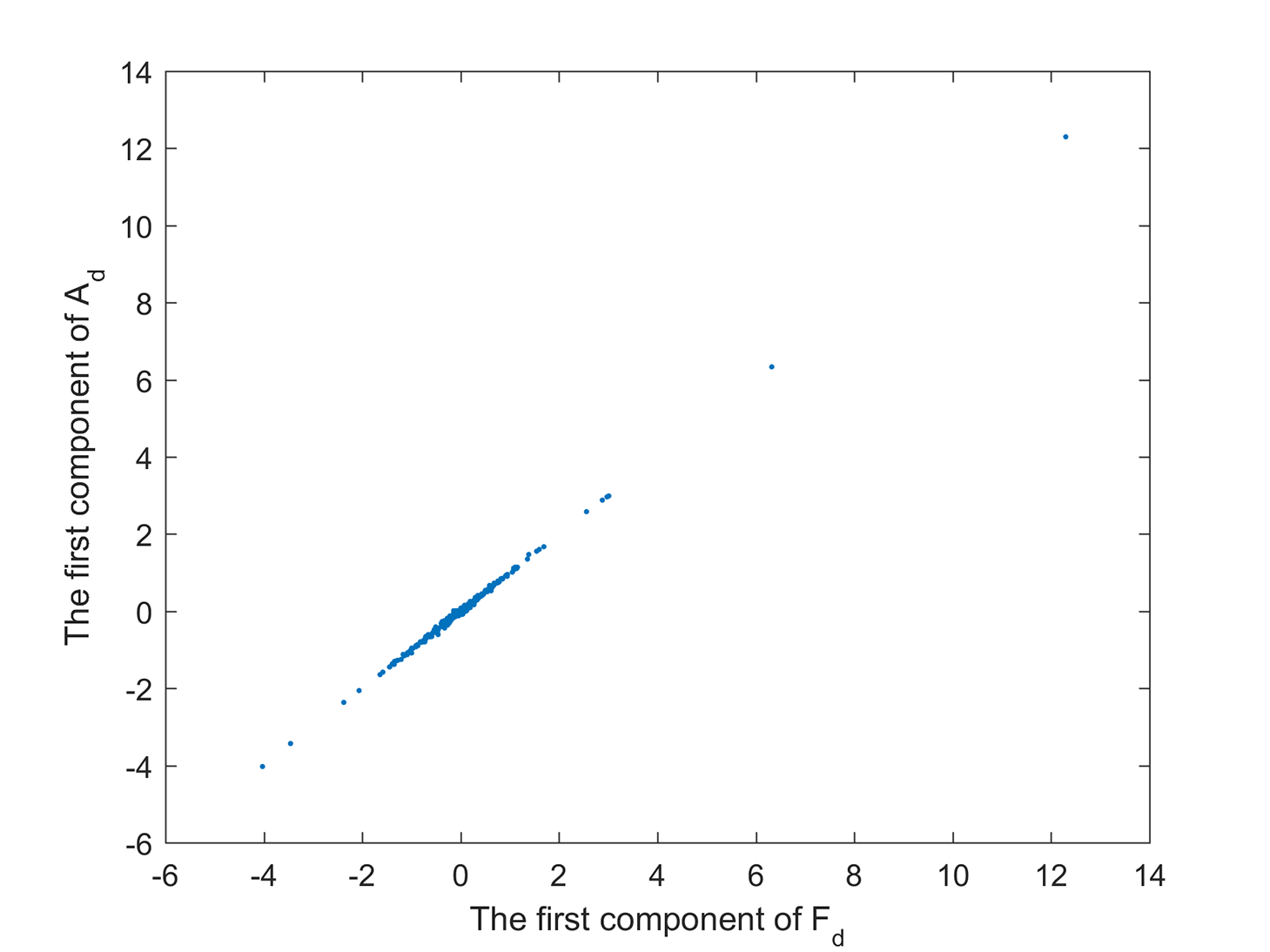

Supplement: Supplementary file 2 [file Image2.TIF]
