# Supplementary material for: GRMDA: Graph Regression for MiRNA-Disease Association Prediction
Source: Front Physiol. 2018 Feb 20;9:92. doi: 10.3389/fphys.2018.00092 (PMC5826195; doi:10.3389/fphys.2018.00092)
Supplement: Supplementary file 4 [file DataSheet1.DOCX]

Supplementary Material

**GRMDA：Graph Regression for MiRNA-Disease Association prediction**

**Xing Chen^1,*^, Jing-Ru Yang^2^, Na-Na Guan^3^, Jian-Qiang Li^3^**

*** Correspondence:**Xing Chen

[xingchen@amss.ac.cn](mailto:xingchen@amss.ac.cn)

# Supplementary Table

**Supplementary Table 1.** We applied GRMDA to prioritize all the candidate miRNA-disease pairs based on all the known miRNA-disease associations in HMDD database as training samples. This prediction result is released for further experimental validation and research.

# Supplementary Figure

**Supplementary Figure 1.** The correlation of the first components of miRNA latent feature matrix $F_{r}$ and miRNA associating matrix $A_{r}$.

**Supplementary Figure 2.** The correlation of the first components of disease latent feature matrix $F_{d}$ and disease associating matrix $A_{d}$.
